# Prospective Comparison of Cognitive MRI-Targeted Biopsy Under Micro-Ultrasound Guidance Versus MRI/Ultrasound Fusion-Guided Transperineal Biopsy for Clinically Significant Prostate Cancer Detection

**DOI:** 10.3390/jcm15197649

**Published:** 2026-10-02

**Authors:** Agostino Fraia, Sara Riolo, Francesco Di Bello, Salvatore Papi, Dario Brunello, Gianluca Nesi, Ivan Di Giulio, Giovanni Costa, Roberto Knez, Tommaso Silvestri, Bernardino de Concilio, Massimiliano Creta, Raffaele Ranavolo, Nicola Longo, Guglielmo Zeccolini, Antonio Celia

**Affiliations:** 1 Department of Urology, San Giuseppe Moscati Hospital, 81031 Aversa, Italy; salvatore.papi@aslcaserta.it (S.P.); raffaele.ranavolo@aslcaserta.it (R.R.); 2 Department of Urology, La Sapienza University, 00185 Rome, Italy; sara.riolo@uniroma1.it (S.R.); gianluca.nesi@hotmail.it (G.N.); 3 Department of Urology, Fundació Puigvert, 08025 Barcelona, Spain; fdibello@fundacio-puigvert.es; 4 Urology Unit, Department of Surgery, Dentistry, Pediatrics and Gynecology, Azienda Ospedaliera Universitaria Integrata, University of Verona, 37126 Verona, Italy; dario.brunello@studenti.univr.it; 5 Department of Urology, San Bassiano Hospital, 36061 Bassano del Grappa, Italy; ivan.digiulio@aulss7.veneto.it (I.D.G.); giovanni.costa1@aulss7.veneto.it (G.C.); roberto.knez@aulss7.veneto.it (R.K.); tommaso.silvestri@aulss7.veneto.it (T.S.); b.deconcilio@aulss7.veneto.it (B.d.C.); guglielmo.zeccolini@aulss7.veneto.it (G.Z.); antonio.celia@aulss7.veneto.it (A.C.); 6 Department of Neurosciences, Reproductive Sciences and Odontostomatology, University of Naples “Federico II”, 80131 Naples, Italy; massimiliano.creta@unina.it (M.C.); nicola.longo@unina.it (N.L.)

**Keywords:** prostate cancer, clinically significant prostate cancer, mpMRI, micro-ultrasound, ExactVu, fusion biopsy, cognitive biopsy, transperineal biopsy

## Abstract

**Background/Objectives**: Multiparametric magnetic resonance imaging (mpMRI)-targeted biopsy has become central to prostate cancer (PCa) diagnosis. While cognitive and software-based fusion targeting have already been compared, the use of high-resolution micro-ultrasound as a real-time guidance platform for cognitive translation of MRI-defined lesions remains incompletely characterized. This study prospectively compared mpMRI-transrectal ultrasound (TRUS) fusion-guided transperineal (TP) biopsy with cognitive MRI-targeted TP biopsy under real-time ExactVu^®^ micro-ultrasound guidance. **Methods**: We conducted a prospective, observational, comparative study including 58 consecutive patients with suspected PCa and at least one lesion on pre-biopsy mpMRI with a Prostate Imaging Reporting and Data System (PI-RADS) score ≥ 3. Twenty-nine patients were included in each group. The analysis used a standardized 16-core TP biopsy set comprising four targeted cores from the MRI-defined index lesion and 12 systematic cores. The primary endpoint was clinically significant PCa (csPCa) detection, defined as International Society of Urological Pathology (ISUP) Grade Group (GG) ≥ 2. **Results**: Overall, PCa detection was 65.5% in the fusion-guided biopsy group and 55.2% in the cognitive MRI-targeted biopsy group (absolute risk difference, 10.3 percentage points; 95% CI, −14.1 to 33.2; *p* = 0.592). csPCa detection was identical in the two groups (37.9% each; absolute risk difference, 0.0 percentage points; 95% CI, −23.6 to 23.6; *p* = 1.000). In exploratory multivariable logistic regression, biopsy technique was not associated with csPCa detection after adjustment (adjusted OR 0.73, 95% CI 0.22–2.40; *p* = 0.605), whereas prostate-specific antigen density (PSAD) showed an association with csPCa detection in the exploratory multivariable model (adjusted OR 1.96 per 0.1-unit increase, 95% CI 1.04–3.71; *p* = 0.039). **Conclusions**: Cognitive MRI targeting under real-time micro-ultrasound guidance showed the same observed csPCa detection rate as software fusion within a standardized targeted-plus-systematic biopsy pathway. Wide confidence intervals preclude conclusions regarding equivalence or non-inferiority.

## 1. Introduction

PCa remains one of the most frequently diagnosed malignancies in men and a major cause of cancer-related morbidity worldwide. Contemporary diagnostic pathways increasingly aim to maximize the detection of csPCa while limiting overdiagnosis of indolent disease, avoiding unnecessary biopsies, reducing biopsy-related morbidity and overtreatment. In current clinical practice, csPCa is most commonly defined as ISUP GG ≥ 2. The European Association of Urology (EAU) recommends mpMRI before prostate biopsy in men with suspected csPCa, confirming the central role of MRI in modern diagnostic work-up [1]. Against this background, the present study examines a specific implementation strategy in a small prospective single-center cohort: cognitive translation of MRI-defined lesions under real-time high-resolution ExactVu^®^ micro-ultrasound guidance without software-based MRI–ultrasound registration. The study was designed to provide exploratory, workflow-specific evidence rather than to re-evaluate the broader comparison between cognitive and software-based fusion targeting.

The evidence supporting an MRI-based pathway is robust. The PRECISION trial showed that an MRI pathway with targeted biopsy improved the detection of clinically significant disease and reduced the diagnosis of clinically insignificant cancer compared with standard systematic biopsy alone [2]. Similarly, the MRI-FIRST study showed that pre-biopsy mpMRI improves the detection of csPCa but does not eliminate the value of systematic cores; the combination of targeted and systematic sampling provided the highest overall diagnostic yield [3]. Consistently, the EAU guideline states that omission of systematic biopsy in biopsy-naïve men may miss a proportion of csPCa, including 16% of ISUP GG ≥ 2 and 18% of ISUP GG ≥ 3 disease [1].

Within MRI-targeted pathways, different targeting techniques are currently available. Among these, mpMRI-TRUS fusion biopsy has become a widely used targeting approach, allowing software-assisted registration of MRI lesions during real-time ultrasound-guided sampling. However, fusion biopsy requires dedicated platforms, lesion contouring, prior MRI segmentation, and operator expertise. Registration errors may arise from contouring inaccuracies, gland deformation, and prostate motion during the procedure. By contrast, cognitive MRI-targeted biopsy relies on pre-biopsy lesion localization and real-time ultrasound guidance without software fusion, potentially simplifying workflow and reducing procedural complexity. Recent comparative studies and meta-analytic data suggest that cognitive and software-fusion MRI-targeted biopsy achieve broadly similar csPCa detection. However, performance can vary according to lesion characteristics and institutional expertise [4,5].

In this context, micro-ultrasound has emerged as a high-resolution real-time imaging modality for prostate biopsy. High-frequency systems such as ExactVu^®^ provide imaging at 29 MHz, allowing detailed visualization of prostatic anatomy during the procedure. Comparative evidence between micro-ultrasound- and MRI-guided biopsy has expanded substantially. The Madrid Protocol evaluated TP biopsy combining ExactVu^®^ micro-ultrasound targeting with mpMRI/ultrasound fusion within the same procedure, suggesting complementary diagnostic information from the two modalities [6]. More recently, the randomized multicenter OPTIMUM trial demonstrated that micro-ultrasound-guided biopsy was non-inferior to MRI/conventional ultrasound fusion-guided biopsy for the detection of ISUP GG ≥ 2 prostate cancer in biopsy-naïve men [7]. Importantly, however, OPTIMUM evaluated micro-ultrasound as an independent targeting modality, rather than as a platform for cognitive translation of MRI-defined targets.

A recent propensity-weighted comparative study similarly reported broadly comparable detection rates between micro-ultrasound- and MRI/ultrasound fusion-guided approaches [8]. Nevertheless, available studies differ in biopsy route, targeting strategy, and the extent to which micro-ultrasound is used independently of, or integrated with, pre-biopsy MRI. The role of ExactVu^®^ specifically as a real-time guidance platform for cognitive localization of MRI-defined lesions therefore remains incompletely characterized. The aim of the present study was to prospectively compare cognitive MRI targeting of the index lesion under real-time ExactVu^®^ micro-ultrasound guidance with mpMRI-TRUS software fusion-guided TP biopsy for the detection of csPCa.

## 2. Materials and Methods

### 2.1. Study Design and Participants

Between June and December 2025, 58 consecutive patients with clinical suspicion of PCa were prospectively included in this observational comparative study conducted at our institution. The relatively small cohort size reflected the predefined single-center recruitment period, during which all consecutive eligible patients were enrolled. Clinical suspicion of PCa was based on elevated prostate-specific antigen (PSA), PSAD, and/or suspicious digital rectal examination findings. All patients underwent pre-biopsy mpMRI for lesion identification and risk stratification. Eligibility criteria included the presence of at least one suspicious lesion on mpMRI (PI-RADS ≥ 3) and suitability for TP prostate biopsy. Biopsy strategy was determined solely by enrollment period: mpMRI-TRUS fusion-guided biopsy was performed during the first period, and cognitive MRI-targeted biopsy was performed during the subsequent period. Twenty-nine participants were included in each group, and no patient-level clinical or radiological criteria influenced biopsy strategy. In both study groups, targeted sampling was directed to the MRI-defined index lesion. According to PI-RADS v2.1, the index lesion was defined as the lesion with the highest PI-RADS category; in patients with multiple lesions sharing the same highest PI-RADS category, the largest lesion was selected. In the fusion group, the index lesion was sampled using dedicated mpMRI-TRUS fusion software, version 3.2. In contrast, in the cognitive ExactVu^®^ (Exact Imaging, Markham, ON, Canada) group, mpMRI findings were used for cognitive localization of the index lesion without software fusion. All procedures were performed transperineally under local anesthesia by urologists experienced in TP prostate biopsy, with the same operators performing procedures using both biopsy techniques. The operators were familiar with mpMRI interpretation and micro-ultrasound guidance.

### 2.2. Workflow

All patients underwent pre-biopsy mpMRI on a 1.5-T Achieva dStream MRI system (Philips Healthcare, Best, The Netherlands). The mpMRI protocol included T2-weighted imaging, diffusion-weighted imaging with apparent diffusion coefficient maps, and dynamic contrast-enhanced sequences. Image interpretation followed PI-RADS version 2.1 criteria [9]. A dedicated uroradiologist with longstanding expertise in prostate MRI interpretation evaluated all mpMRI examinations. The PI-RADS category, size, and zonal location of the MRI-defined index lesion were recorded, and the index lesion was considered for all lesion-level analyses.

#### 2.2.1. mpMRI-TRUS Fusion Biopsy

In the fusion biopsy group, procedures were performed using a MyLab™ Eight ultrasound system (Esaote S.p.A., Genoa, Italy). A single dedicated uroradiologist reviewed the mpMRI for procedural planning and manually contoured the MRI-defined index lesion slice by slice on T2-weighted images before transfer to the BiopSee^®^ platform, version 3.2 (MedCom GmbH, Darmstadt, Germany). The images were subsequently exported from the hospital picture archiving and communication system to the BiopSee^®^ platform for software-assisted targeting.

#### 2.2.2. Cognitive MRI-Targeted Biopsy

In the cognitive biopsy group, the operating urologist reviewed the pre-biopsy mpMRI images before the procedure to assess lesion location, size, PI-RADS score, and relevant anatomical landmarks of the index lesion. MRI-derived information on lesion laterality, zonal location, apical–midgland–basal position, depth, and relationship to recognizable prostatic landmarks was used to cognitively reproduce the expected position of the index lesion during real-time micro-ultrasound imaging. Biopsy was then performed using the ExactVu^®^ platform (Exact Imaging, Markham, ON, Canada), without software-based MRI–ultrasound fusion. Micro-ultrasound was used as the real-time imaging modality for cognitive localization and targeting of the MRI-defined index lesion rather than as an independent lesion-detection strategy. Prostate risk identification using micro-ultrasound (PRI-MUS) scores was not included in the present analysis. No additional targets or targeted cores were selected solely based on micro-ultrasound appearance.

### 2.3. Biopsy Technique

All biopsy procedures were performed transperineally with the patient in the lithotomy position. Bowel preparation consisted of a cleansing enema administered the evening before the procedure. Antibiotic prophylaxis consisted of oral trimethoprim–sulfamethoxazole 160/800 mg administered on the day of the procedure in accordance with local institutional protocols. After perineal shaving and sterile preparation of the perineal skin with povidone–iodine (Braunol^®^; B. Braun Milano S.p.A., Milan, Italy), ultrasound gel was applied to the probe, which was then covered with a disposable probe cover and inserted transrectally. Local anesthesia was administered bilaterally at the prostatic apex using a 25-gauge spinal needle advanced through the perineal tissues, with 10 mL of lidocaine injected on each side. Biopsy sampling was performed using a coaxial technique with an 18-gauge needle cannula and an 18-gauge, 250 mm biopsy needle (HS NOTA18250; HS Hospital Service S.p.A., Aprilia, Italy). One cannula was inserted per lobe through the perineal skin, approximately 1 cm lateral to the median raphe and 1 cm above the anal verge. Four targeted cores were obtained from the MRI-defined index lesion, followed by systematic sampling with six cores per lobe.

### 2.4. Data Collection

Prospective data collection included demographic, clinical, radiological, biopsy-related, and pathological variables for all patients. Demographic and clinical variables included age, age-adjusted Charlson Comorbidity Index (CCI) [10], baseline PSA, PSAD, use of alpha-blockers and 5-alpha-reductase inhibitors, direct oral anticoagulant therapy, antiplatelet therapy, and biopsy setting (initial vs. repeat biopsy). Pre-procedural radiological variables included prostate volume, PI-RADS category, and the anatomical location of the index lesion, as well as the presence of seminal vesicle invasion or extraprostatic extension on mpMRI. Biopsy-related variables included the total number of cores analyzed, the number of targeted and systematic cores, the total number of positive cores, and the number of positive cores by targeted and systematic sampling. Pathological variables included ISUP GG [11]. Biopsy specimens were evaluated by a single dedicated genitourinary pathologist with longstanding expertise in prostate pathology, according to the ISUP GG system. The pathologist was blinded to the biopsy technique. Post-biopsy adverse events occurring within 30 days after the procedure, including urinary tract infection (UTI), fever, urinary retention requiring catheterization, hematuria requiring medical evaluation, septic complications, and hospital admission, were prospectively recorded.

### 2.5. Statistical Analysis

All 58 enrolled patients were included in the statistical analysis. The study cohort comprised consecutive eligible patients recruited during the predefined study period. No formal a priori sample-size or power calculation was performed; the sample size reflected all consecutive eligible patients enrolled during the predefined study period. Continuous variables were reported as the median and interquartile range (IQR) and compared using the Mann–Whitney U test. Categorical variables were expressed as numbers and percentages and compared using Fisher’s exact test or the chi-square test, as appropriate; exact tests were preferred when expected cell counts were small. For multicategory contingency tables with sparse expected cell counts, the Fisher–Freeman–Halton exact test was used. The age-adjusted CCI was analyzed as a four-category variable (1, 2, 3, and >3), whereas the overall PI-RADS distribution was compared across PI-RADS categories 3, 4, and 5. The age-adjusted CCI was collected to characterize baseline comorbidity burden. Given the limited number of csPCa events, it was not included in the exploratory multivariable model, which was deliberately restricted to variables considered more directly relevant to csPCa detection and potential confounding of the technique comparison.

The primary endpoint was csPCa detection on combined targeted plus systematic biopsy, defined as ISUP GG ≥ 2. Effect estimates were reported as odds ratios (ORs) and absolute risk differences with 95% confidence intervals (CIs). CIs for absolute risk differences between independent proportions were calculated using the Newcombe score method based on Wilson score intervals without continuity correction. Univariable logistic regression analyses were performed to evaluate associations with csPCa detection, including age, PSA, PSAD, prostate volume, PI-RADS category (≥ 4 vs. 3), biopsy setting (repeat vs. initial biopsy), and biopsy technique. Continuous variables were modeled per clinically meaningful unit where appropriate, including PSAD per 0.1-unit increase. An exploratory multivariable logistic regression model was fitted including biopsy technique, PI-RADS category (≥4 vs. 3), PSAD, and biopsy setting (repeat vs. initial biopsy). Given the limited number of events, the multivariable model was considered exploratory and restricted to clinically relevant variables. Variables were selected on the basis of clinical relevance and potential confounding rather than statistical significance in univariable analysis. To assess the robustness of the primary comparison given the baseline imbalance in MRI suspicion categories, a post hoc sensitivity analysis was performed after restricting the cohort to patients with PI-RADS ≥ 4 index lesions. All statistical tests were two-sided, and *p*-values < 0.05 were considered statistically significant. Statistical analyses were performed using IBM SPSS Statistics, version 32.0.0 (IBM Corp., Armonk, NY, USA).

## 3. Results

The study included 58 patients. The median age of the overall cohort was 68.0 years (IQR 64.0–74.0), with no statistically significant difference between the fusion and cognitive groups (67.0 years, IQR 63.0–71.0 vs. 70.0 years, IQR 66.0–75.0; *p* = 0.072). Age-adjusted CCI distribution was significantly different between the two groups (*p* = 0.001), with a higher proportion of patients with a CCI score of 3 in the fusion group and a higher proportion with a score of 2 in the cognitive group. Median PSA values did not differ between groups (6.2 vs. 6.2 ng/mL; *p* = 0.597), nor did median PSAD values (0.14 vs. 0.12 ng/mL/mL; *p* = 0.460). Table 1 summarizes baseline demographic and clinical characteristics of the overall cohort and the two study groups. No cases of UTI, fever, or urinary retention requiring catheterization were recorded in either group. Furthermore, no major post-biopsy complications, including hematuria requiring medical evaluation, septic events, or hospital admission, occurred.

### 3.1. MRI Findings

Table 2 summarizes baseline MRI characteristics for the overall cohort and the two study groups. Overall, the median prostate volume on MRI was significantly lower in the fusion group compared to the cognitive group (40.0 vs. 54.0 mL; *p* = 0.035). The overall distribution of PI-RADS categories also differed significantly between groups (*p* = 0.013), with a higher proportion of PI-RADS 3 lesions and a lower proportion of PI-RADS 4 lesions in the fusion group. Accordingly, PI-RADS ≥ 4 index lesions were less frequent in the fusion group than in the cognitive group (69.0% vs. 96.6%; *p* = 0.012). The median index lesion size was 10.1 mm (IQR 8.0–14.8). Although index lesions were larger in the fusion group than in the cognitive group (12.0 vs. 9.0 mm), the difference was not statistically significant (*p* = 0.123). Index lesions were more frequently located in the peripheral zone than in the transition zone in both groups. No patient showed seminal vesicle invasion or extraprostatic extension on pre-biopsy mpMRI.

### 3.2. Histopathological Findings

**Figure 1:**
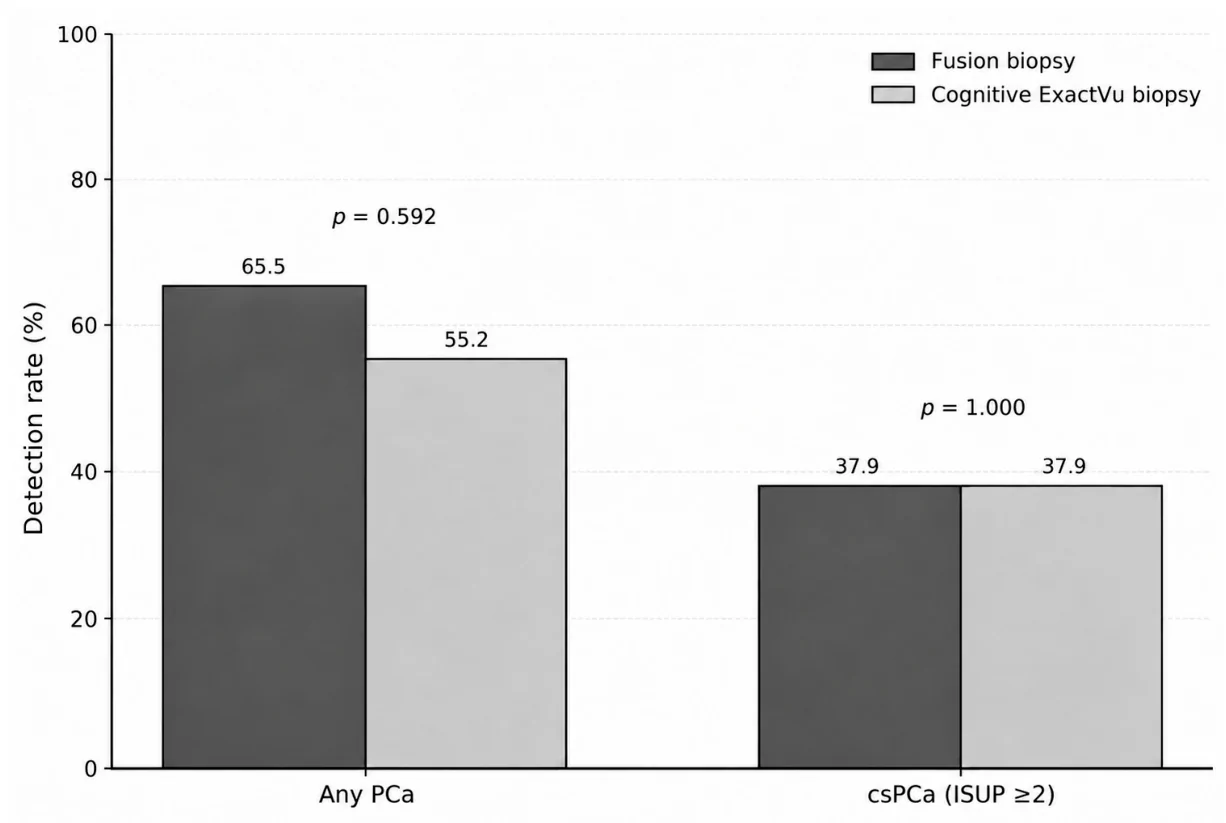
Comparative detection rates of PCa and csPCa (defined as ISUP Grade Group ≥ 2) in the fusion biopsy and cognitive ExactVu biopsy groups.

Table 3 summarizes the histopathological findings. Overall, PCa was detected in 35 patients (60.3%), while csPCa, defined as ISUP GG ≥ 2, was identified in 22 patients (37.9%). ISUP GG 1 was found in 13 patients (22.4%), ISUP GG 2 in 16 patients (27.6%), ISUP GG 3 in four patients (6.9%), and ISUP GG 4–5 in 2 patients (3.4%). No statistically significant difference was observed between the two groups in either overall PCa detection (65.5% vs. 55.2%; absolute risk difference 10.3 percentage points, 95% CI −14.1 to 33.2; *p* = 0.592) or csPCa detection (37.9% vs. 37.9%; absolute risk difference 0.0 percentage points, 95% CI −23.6 to 23.6; *p* = 1.000), as shown in Figure 1. In a post hoc sensitivity analysis restricted to patients with PI-RADS ≥ 4 index lesions, csPCa was detected in 50.0% of patients in the fusion group and 35.7% in the cognitive biopsy group (*p* = 0.382). Given the limited subgroup size, this analysis was considered exploratory and was not interpreted as evidence of similar or equivalent performance (Supplementary Table S1).

### 3.3. Factors Associated with csPCa Detection

An exploratory logistic regression analysis was performed to evaluate factors associated with csPCa, defined as ISUP GG ≥ 2. In the univariable analysis, none of the evaluated variables reached statistical significance, although PSA and PSAD showed non-significant trends toward association with csPCa detection. In the multivariable model including biopsy technique, PI-RADS category (≥4 vs. 3), PSAD, and biopsy setting (repeat vs. initial biopsy), PSAD showed an association with csPCa detection after adjustment for the variables included in the exploratory multivariable model (adjusted OR 1.96 per 0.1 increase, 95% CI 1.04–3.71; *p* = 0.039). Conversely, biopsy technique was not associated with csPCa detection after adjustment (adjusted OR 0.73, 95% CI 0.22–2.40; *p* = 0.605). Table 4 reports the complete results of the univariable and multivariable analyses.

## 4. Discussion

In this prospective comparative study, cognitive MRI targeting of the index lesion under real-time ExactVu^®^ micro-ultrasound guidance yielded the same observed csPCa detection rate as mpMRI-TRUS software fusion-guided biopsy within a standardized TP targeted-plus-systematic biopsy pathway. Overall PCa detection also did not differ significantly between groups, and biopsy technique was not associated with csPCa detection after adjustment in the exploratory multivariable analysis. Conversely, PSAD showed an association with csPCa detection after adjustment for PI-RADS category, biopsy setting, and biopsy technique. Importantly, although the observed csPCa detection rates were identical, the wide confidence interval around the between-group difference precludes conclusions regarding equivalence or non-inferiority. More broadly, the biological heterogeneity of prostate cancer across the disease continuum reinforces the importance of accurate disease characterization [12].

This interpretation is coherent with the broader evolution of prostate cancer diagnosis. Contemporary guideline-based practice places pre-biopsy mpMRI at the center of the diagnostic work-up and recognizes MRI-targeted biopsy as a key component of csPCa detection [13]. The STHLM3-MRI trial confirmed the value of MRI-based risk-adapted pathways in improving clinically relevant cancer detection while reducing unnecessary biopsy [14]. At the same time, biopsy performance cannot be reduced to targeting technology alone. In the work by Ahdoot et al., combined MRI-targeted and systematic biopsy improved diagnostic accuracy compared with either approach alone in men with MRI-visible lesions [15]. Against this background, our findings suggest that the performance of the overall diagnostic pathway, including patient selection, MRI interpretation, and integration of targeted and systematic sampling, may influence diagnostic yield alongside the choice of targeting technique. Previous prospective urological studies have similarly evaluated systematically collected clinical and imaging-derived variables in relation to clinically relevant outcomes [16,17].

The present study addresses a different question from that examined in the IMAGINATION trial. IMAGINATION provided randomized multicenter evidence that conventional cognitive fusion, based on visual estimation and mental registration of MRI findings during ultrasound-guided biopsy, is non-inferior to software-based fusion for targeted-biopsy csPCa detection. In that multicenter randomized trial, cognitive targeting was performed without fusion-workstation assistance using conventional ultrasound guidance, whereas the present study specifically evaluated a hybrid MRI-to-micro-ultrasound workflow in which a previously identified MRI index lesion was cognitively translated and targeted under real-time high-resolution ExactVu^®^ micro-ultrasound guidance. Micro-ultrasound was therefore used as an anatomical guidance platform rather than as an independent PRI-MUS-based lesion-detection modality [18].

Accordingly, the present study was not intended to re-examine the general non-inferiority of cognitive versus software-based fusion, which has now been addressed by IMAGINATION. Rather, it provides implementation-specific prospective data on whether MRI-defined lesions can be targeted cognitively using micro-ultrasound as the real-time imaging interface without MRI–ultrasound software registration. This specific workflow was not evaluated in IMAGINATION [18]. In addition, our study applied a standardized transperineal protocol with four targeted and 12 systematic cores, with the same operators performing both biopsy strategies, thereby providing information on the performance of this MRI-to-micro-ultrasound approach within a consistent clinical pathway. These findings should nevertheless be regarded as exploratory given the small, single-center, non-randomized cohort.

Our findings should also be interpreted in the context of the expanding evidence on micro-ultrasound-guided prostate biopsy. The randomized multicenter OPTIMUM trial demonstrated non-inferiority of micro-ultrasound-guided biopsy compared with MRI/conventional ultrasound fusion-guided biopsy for detecting ISUP GG ≥2 disease in biopsy-naïve men [7]. Other comparative studies have similarly reported broadly similar diagnostic performance between micro-ultrasound- and MRI/ultrasound fusion-guided approaches [19,20,21,22]. However, these studies have predominantly evaluated micro-ultrasound as an independent lesion-detection and targeting modality or within dedicated fusion workflows. By contrast, in the present study, ExactVu^®^ was used as the real-time guidance platform for cognitive targeting of an MRI-defined index lesion, without software-based image registration.

Another clinically relevant observation is that PSAD showed an association with csPCa detection after adjustment in the exploratory multivariable model, despite not reaching statistical significance on univariable analysis. This finding may support the established role of PSAD as an important component of risk stratification within MRI-based diagnostic pathways. However, the model was exploratory and based on only 22 csPCa events; therefore, this association requires confirmation in larger cohorts.

From an implementation perspective, the present study also has practical value. Fusion biopsy requires dedicated software, lesion contouring, image registration, and a more elaborate workflow involving radiologist input and platform preparation. By contrast, the cognitive ExactVu^®^ workflow relied on pre-procedural MRI review and real-time micro-ultrasound guidance. This may offer a simpler workflow in selected settings; however, the present study did not formally assess procedural time, costs, resource utilization, or operator workload. Therefore, no conclusions regarding economic or organizational superiority can be drawn from these data [19,20,21].

The present study has several limitations. First, the sample size was modest, resulting in wide confidence intervals around the between-group estimates; accordingly, the study should be considered exploratory and was not designed to establish equivalence or non-inferiority. Second, the study was non-randomized, and the two biopsy strategies were applied sequentially based solely on enrollment period. Although no patient-level clinical or radiological criteria determined biopsy strategy, temporal effects, including changes in operator experience or clinical workflow, cannot be excluded. Accordingly, the observed between-group findings should be interpreted as an observational comparison of two sequentially implemented biopsy strategies rather than as a causal estimate of the effect of the targeting technique. Third, baseline imbalances were present, particularly in prostate volume and PI-RADS distribution. Because biopsy strategy was determined solely by enrollment period, the PI-RADS imbalance reflected differences in case mix between the two sequential recruitment periods. Although the exploratory multivariable model included PI-RADS category, residual confounding remains possible. Moreover, the lack of statistical significance in the PI-RADS ≥ 4 sensitivity analysis should not be interpreted as evidence of similar or equivalent performance. These baseline imbalances may have influenced the observed detection rates independently of the biopsy strategy, particularly given the higher prevalence of PI-RADS ≥ 4 lesions in the cognitive biopsy group. Fourth, micro-ultrasound was used for real-time cognitive targeting of MRI-defined lesions rather than as an independent lesion-detection strategy, and PRI-MUS scores were not prospectively recorded. Therefore, the present findings should not be extrapolated to stand-alone micro-ultrasound biopsy or interpreted as an assessment of PRI-MUS-based lesion detection. Because the primary endpoint was based on combined targeted and systematic sampling, the present study evaluates the diagnostic performance of the overall biopsy strategies rather than the isolated performance of the two targeting techniques. In addition, the two study strategies differed not only in targeting method but also in ultrasound platform and MRI-to-target workflow; therefore, the present comparison cannot isolate the independent contribution of cognitive versus software-assisted registration. Clinically significant prostate cancer was defined as ISUP GG ≥ 2. However, GG2 disease is heterogeneous, and current active-surveillance protocols may include selected patients with favorable GG2 features according to different eligibility criteria. Because the percentage of Gleason pattern 4 was not prospectively recorded in our cohort, further stratification of GG2 tumors according to pattern 4 burden could not be performed [1]. Given the limited sample size, the absence of serious complications in this cohort should not be interpreted as evidence of a low incidence of uncommon adverse events or as a comparative assessment of procedural safety. Finally, biopsy pathology rather than whole-mount prostatectomy or long-term clinical follow-up served as the reference standard, as is common in comparative biopsy studies [1,2].

## 5. Conclusions

In conclusion, this study evaluated the cognitive translation of MRI-defined index lesions under real-time ExactVu^®^ micro-ultrasound guidance within a transperineal targeted-plus-systematic biopsy pathway. Although the two groups showed the same observed csPCa detection rate, the small sample size, sequential non-randomized design, baseline imbalances, and wide confidence intervals preclude conclusions regarding equivalence or non-inferiority. Further prospective multicenter studies are needed to validate the diagnostic performance and clinical role of this specific MRI-to-micro-ultrasound workflow.

## Figures and Tables

**Table 1:** Demographic and preoperative clinical data.

Variable	Overall (*n* = 58)	FUS-TB (*n* = 29)	COG-TB (*n* = 29)	*p*-Value
Age-adjusted CCI, points (%)				
1	2 (3.4)	1 (3.4)	1 (3.4)	
2	10 (17.2)	0 (0.0)	10 (34.5)	
3	27 (46.6)	18 (62.1)	9 (31.0)	
>3	19 (32.8)	10 (34.5)	9 (31.0)	
Overall CCI distribution				0.001
PSA, ng/mL, median (IQR)	6.2 (3.9–8.7)	6.2 (4.1–7.7)	6.2 (3.8–9.4)	0.597
PSAD, ng/mL/mL, median (IQR)	0.13 (0.06–0.21)	0.14 (0.11–0.20)	0.12 (0.06–0.21)	0.460
Alpha-blocker use, *n* (%)	10 (17.2)	5 (17.2)	5 (17.2)	1.000
5-ARI use, *n* (%)	5 (8.6)	1 (3.4)	4 (13.8)	0.352
DOAC therapy, *n* (%)	6 (10.3)	2 (6.9)	4 (13.8)	0.670
APT, *n* (%)	9 (15.5)	3 (10.3)	6 (20.7)	0.470
Initial biopsy, *n* (%)	32 (55.2)	14 (48.3)	18 (62.1)	0.429
Repeat biopsy, *n* (%)	26 (44.8)	15 (51.7)	11 (37.9)	0.429

Abbreviations: 5-ARI, 5α-Reductase Inhibitor; APT, Antiplatelet Therapy; CCI, Charlson Comorbidity Index; COG-TB, Cognitive Targeted Biopsy; DOAC, Direct Oral Anticoagulant; FUS-TB, Fusion Targeted Biopsy; IQR, Interquartile Range; PSA, Prostate-Specific Antigen; PSAD, PSA Density.

**Table 2:** MRI findings.

Variable	Overall (*n* = 58)	FUS-TB (*n* = 29)	COG-TB (*n* = 29)	*p*-Value
Prostate volume, mL, median (IQR)	48.5 (35.0–59.4)	40.0 (31.0–54.0)	54.0 (40.0–62.0)	0.035
Index lesion size, mm, median (IQR)	10.1 (8.0–14.8)	12.0 (9.0–15.0)	9.0 (8.0–14.0)	0.123
PI-RADS 3, *n* (%)	10 (17.2)	9 (31.0)	1 (3.4)	
PI-RADS 4, *n* (%)	37 (63.8)	14 (48.3)	23 (79.3)	
PI-RADS 5, *n* (%)	11 (19.0)	6 (20.7)	5 (17.2)	
PI-RADS distribution				0.013
PI-RADS ≥ 4, *n* (%)	48 (82.8)	20 (69.0)	28 (96.6)	0.012
Peripheral zone ROI, *n* (%)	40 (69.0)	17 (58.6)	23 (79.3)	0.155
Transition zone ROI, *n* (%)	18 (31.0)	12 (41.4)	6 (20.7)	0.155

Abbreviations: COG-TB, Cognitive Targeted Biopsy; FUS-TB, Fusion Targeted Biopsy; IQR, Interquartile Range; PI-RADS, Prostate Imaging Reporting and Data System; ROI, Region of Interest.

**Table 3:** Histopathological findings.

Variable	Overall (*n* = 58)	FUS-TB (*n* = 29)	COG-TB (*n* = 29)	*p*-Value
Any PCa, *n* (%)	35 (60.3)	19 (65.5)	16 (55.2)	0.592
ISUP GG 1, *n* (%)	13 (22.4)	8 (27.6)	5 (17.2)	
ISUP GG ≥ 2, *n* (%)	22 (37.9)	11 (37.9)	11 (37.9)	1.000
ISUP GG 2, *n* (%)	16 (27.6)	9 (31.0)	7 (24.1)	
ISUP GG 3, *n* (%)	4 (6.9)	1 (3.4)	3 (10.3)	
ISUP GG 4–5, *n* (%)	2 (3.4)	1 (3.4)	1 (3.4)	

Abbreviations: COG-TB, Cognitive Targeted Biopsy; FUS-TB, Fusion Targeted Biopsy; GG, Grade Group; ISUP, International Society of Urological Pathology; PCa, Prostate Cancer.

**Table 4:** Univariable and multivariable logistic regression analyses of factors associated with csPCa detection.

Variable	OR	95% CI	*p*-Value	aOR	95% CI	*p*-Value
Age, per year	0.96	0.89–1.03	0.243	—	—	—
PSA, ng/mL	1.12	0.98–1.28	0.084	—	—	—
PSAD, per 0.1 increase	1.62	0.94–2.80	0.085	1.96	1.04–3.71	0.039
Prostate volume, mL	0.99	0.98–1.01	0.553	—	—	—
PI-RADS ≥ 4 vs. 3	2.86	0.55–14.91	0.213	5.14	0.70–37.91	0.109
Repeat vs. initial biopsy	0.57	0.19–1.69	0.313	0.61	0.18–2.04	0.425
Cognitive vs. fusion biopsy	1.00	0.35–2.89	1.000	0.73	0.22–2.40	0.605

Abbreviations: aOR, adjusted Odds Ratio for the multivariable analysis; CI, Confidence Interval; csPCa, clinically significant Prostate Cancer; OR, Odds Ratio for the univariable analysis; PI-RADS, Prostate Imaging Reporting and Data System; PSA, Prostate-Specific Antigen; PSAD, Prostate-Specific Antigen density.

## Data Availability

The datasets generated during and/or analyzed during the current study are available from the corresponding author on reasonable request.

## Supplementary Materials

The following supporting information can be downloaded at https://www.mdpi.com/article/10.3390/jcm15197649/s1. Table S1: Sensitivity analysis of PCa and csPCa detection rates restricted to patients with PI-RADS ≥ 4 index lesions.

## Abbreviations

The following abbreviations are used in this manuscript:

5-ARI	5α-Reductase Inhibitor
APT	Antiplatelet Therapy
CCI	Charlson Comorbidity Index
COG-TB	Cognitive Targeted Biopsy
csPCa	clinically significant Prostate Cancer
DOAC	Direct Oral Anticoagulant
EAU	European Association of Urology
FUS-TB	Fusion Targeted Biopsy
GG	Grade Group
ISUP	International Society of Urological Pathology
mpMRI	multiparametric Magnetic Resonance Imaging
MRI	Magnetic Resonance Imaging
PCa	Prostate Cancer
PI-RADS	Prostate Imaging Reporting and Data System
PRI-MUS	Prostate Risk Identification using Micro-Ultrasound
PSA	Prostate-Specific Antigen
PSAD	PSA density
ROI	Region of Interest
TP	Transperineal
TRUS	Transrectal Ultrasound
UTI	Urinary Tract Infection
