# Supplementary material for: Prospective Comparison of Cognitive MRI-Targeted Biopsy Under Micro-Ultrasound Guidance Versus MRI/Ultrasound Fusion-Guided Transperineal Biopsy for Clinically Significant Prostate Cancer Detection
Source: J Clin Med. 2026 Oct 2;15(19):7649. doi: 10.3390/jcm15197649 (PMC13666145; doi:10.3390/jcm15197649)
Supplement: Supplementary file 1 [file jcm-15-07649-s001.zip › jcm-4563892-supplementary.pdf]

Supplementary Table S1. Sensitivity analysis of PCa and csPCa detection rates restricted to patients with PI-RADS ≥ 4 index lesions.

Variable	Overall (n = 48)	FUS-TB (n = 20)	COG-TB (n = 28)	p-value
Any PCa, n (%)	30 (62.5)	15 (75.0)	15 (53.6)	0.226
csPCa (ISUP GG ≥2), n (%)	20 (41.7)	10 (50.0)	10 (35.7)	0.382

Abbreviations: COG-TB, Cognitive Targeted Biopsy; csPCa, clinically significant Prostate Cancer; FUS-TB, Fusion Targeted Biopsy; GG, Grade Group; ISUP, International Society of Urological Pathology; PCa, Prostate Cancer; PI-RADS, Prostate Imaging Reporting and Data System.
